# Treg–tissue cell interactions in repair and regeneration

**DOI:** 10.1084/jem.20231244

**Published:** 2024-04-26

**Authors:** Lucas F. Loffredo, Thomas M. Savage, Olivia R. Ringham, Nicholas Arpaia

**Affiliations:** 1 Department of Microbiology and Immunology, Columbia University, New York, NY, USA; 2 Herbert Irving Comprehensive Cancer Center, Columbia University, New York, NY, USA

## Abstract

Regulatory T (Treg) cells are classically known for their critical immunosuppressive functions that support peripheral tolerance. More recent work has demonstrated that Treg cells produce pro-repair mediators independent of their immunosuppressive function, a process that is critical to repair and regeneration in response to numerous tissue insults. These factors act on resident parenchymal and structural cells to initiate repair in a tissue-specific context. This review examines interactions between Treg cells and tissue-resident non-immune cells—in the context of tissue repair, fibrosis, and cancer—and discusses areas for future exploration.

## Teaser

Treg cells are classically known for their critical immunosuppressive functions. More recent work demonstrates that Treg cells produce mediators that facilitate tissue repair. This review examines the interactions between Treg cells and tissue-specific non-immune cells that mediate repair and regeneration following sterile or infectious damage.

## Introduction

Regulatory T (Treg) cells are a specialized CD4^+^ T cell subset first identified for their critical immunosuppressive functions (Josefowicz et al., 2012; Sakaguchi et al., 2020). The absence of Treg cells, most definitively seen in Scurfy mice, results in uncontrolled inflammation, tissue damage, and death—a phenotype that can be rescued by the transfer of CD4^+^ T cells overexpressing *Foxp3* (Fontenot et al., 2003; Hori et al., 2003). The demonstration of Foxp3 as the lineage-defining transcription factor of Treg cells resulted in a close study of the critical roles that Treg cells play in maintaining immunological tolerance to self-antigens and to innocuous environmental antigens such as food and commensal bacteria (Josefowicz et al., 2012). Extensive study has identified the immunosuppressive mediators—and the mechanisms through which they act—that enable Treg cell function, including cytokines such as IL-10 and TGF-β, cell surface proteins such as CTLA-4, sequestration of the critical T cell cytokine IL-2 through constitutive CD25 expression, and microenvironmental metabolic changes, such as conversion of proinflammatory ATP to anti-inflammatory AMP via expression of CD73 and CD39, among other processes (Dikiy and Rudensky, 2023; Josefowicz et al., 2012). The effect of these mediators on other immune cells, including macrophages, dendritic cells, and T cells, represents the canonical function of Treg cells and remains under intense investigation (Dikiy and Rudensky, 2023).

**Figure 1.**
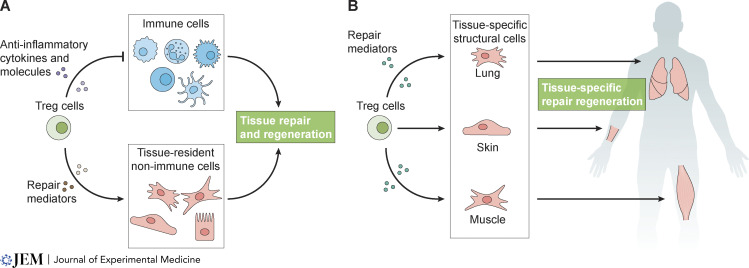
**Function of Treg cells in tissue repair. (A)** Treg cells mediate tissue repair and regeneration via the production of anti-inflammatory molecules that reduce inflammation (top) and through the production of mediators that influence tissue-resident non-immune cells (bottom)—a function that is distinct from immunosuppression. **(B)** Treg cell–derived mediators are sensed by unique tissue-specific parenchymal and structural cells, which generate a tissue- and context-specific repair program.

Beyond their effect on modulating immune responses, more recent work has shown that Treg cells can play critical roles in tissue repair and regeneration. This can occur via protection of tissue stem cell niches from inflammatory activity (Fujisaki et al., 2011) or by direct signaling to non-immune tissue cells and/or stem cells, which is the subject of this review (Fig. 1 A). Critically, the production of non-immunomodulatory mediators is distinct from Treg cell immunosuppressive function (Arpaia et al., 2015). This suggests a model in which Treg cells promote tissue repair and regeneration through distinct modalities; namely, indirectly by limiting collateral tissue damage through immunosuppressive molecules that act on immune cells, and directly through the production of tissue factors that are sensed by mesenchymal, epithelial, and endothelial cells (Fig. 1 A). In most damage resolution scenarios, both of these mechanisms of action are likely at play; however, many of the reports we review herein effectively isolate these individual processes through elegant experimentation. Crucially, the sensing of Treg cell–derived mediators by specialized tissue-resident non-immune cells allows a common input to generate a context- and tissue-specific program that is unique and critical for regeneration and repair (Fig. 1 B). In this review, we will discuss the interplay of Treg cells with tissue-specific parenchymal and structural cells and their emerging role in tissue protection.

## Treg cell–tissue cell intercommunication in tissue regeneration

**Figure 2.**
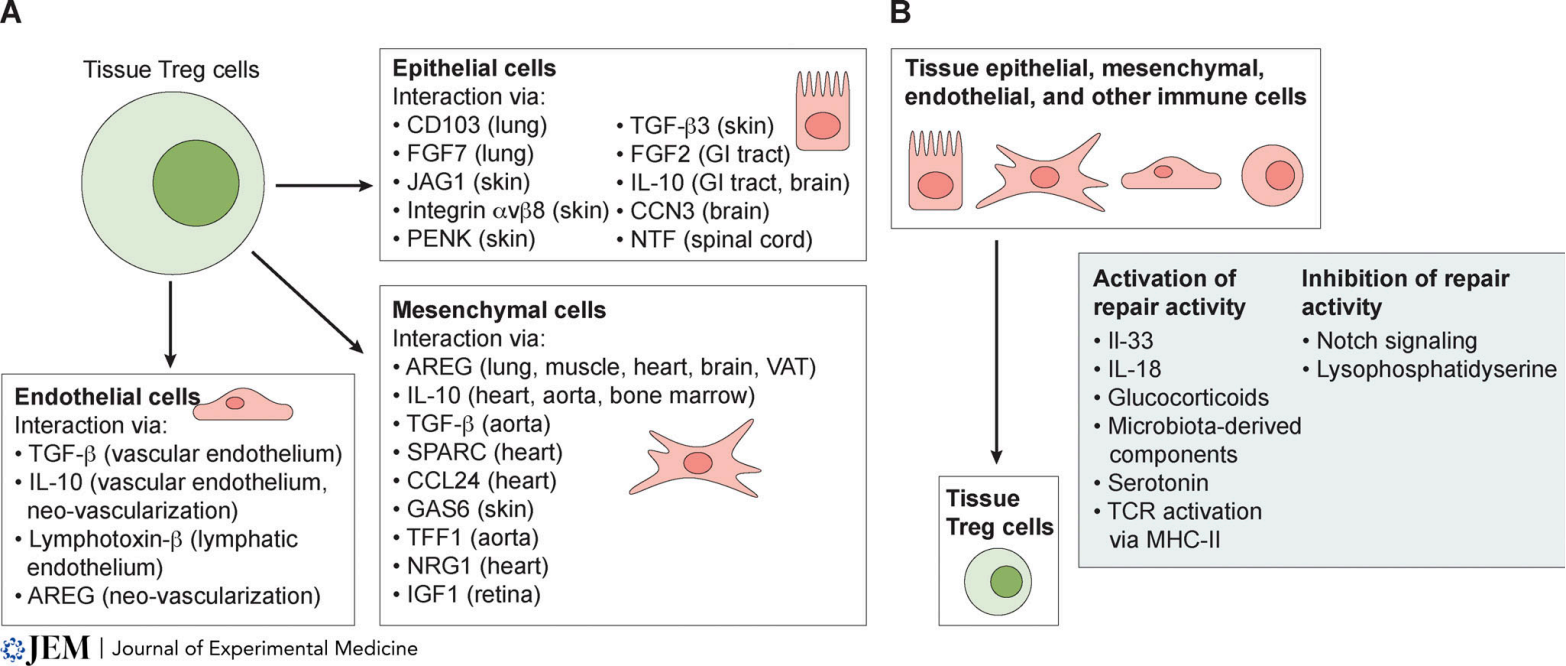
**Mediators of Treg cell interactions with tissue cells. (A)** Treg cell–derived factors shown to mediate communication with each type of tissue cell, as well as the organ(s) where these effects were studied. **(B)** Tissue-derived mediators shown to influence Treg cell reparative programs.

Several recent reviews discuss the roles of Treg cells during homeostasis and disease in various non-lymphoid tissue sites (Astarita et al., 2023; Boothby et al., 2020; Estrada Brull et al., 2022; Jovisic et al., 2023; Panduro et al., 2016; Zhang et al., 2017); however, none have focused specifically on how Treg cells communicate with other tissue cell types. Thus, herein we review existing literature reporting interactions between Treg cells and epithelial, mesenchymal, or endothelial cells (hereafter referred to as “tissue cells”) that mediate regeneration (Fig. 2 A), as well as the tissue cell–derived signals that modulate Treg cell repair activity (Fig. 2 B).

### Treg cell–epithelial cell interactions

There have been several reports investigating Treg cell interactions with epithelial cells across multiple organ systems and in several disease states. We first discuss these examples in the context of experimental lung disease.

A seminal report by the King group (D’Alessio et al., 2009) is the first to our knowledge indicating that Treg cells may promote tissue reparative processes. The authors observed deficient resolution of tissue damage following acute lung injury (ALI) in the absence of lymphocytes, a phenotype that could be rescued by adoptive transfer of CD4^+^CD25^+^ splenic T cells, but not other T cell subsets; however, this was attributed to Treg cell–mediated effects on alveolar macrophages, not to their direct action on tissue cells. In a follow-up study, the authors imply that lung Treg cells reduce the production of CXCL12 by lung epithelial cells (LECs) (Garibaldi et al., 2013).

Additional reports by this group demonstrated direct Treg cell–LEC communication in this context. One such report explored the role of CD103 (*Itgae*, an integrin receptor for E-cadherin) expression by lung Treg cells (Mock et al., 2014). In this study, the proliferation of E-cadherin^+^ LECs was shown to correlate with an increase in CD103^+^ Treg cells in the lung following ALI, and LEC proliferation was decreased upon Treg cell depletion. This was also tested in a Treg cell–replete environment by adoptively transferring wild type or *Itgae*^*−/−*^ Treg cells into *Rag1*^*−/−*^ mice, showing decreased LEC proliferation in the latter. In a later report, it was found that Treg cell–derived FGF7 (KGF) promotes LEC proliferation in both the LPS ALI and pneumectomy contexts (Dial et al., 2017). These reports also showed that *Itgae*^*−/−*^ and *Fgf7*^*−/−*^ Treg cells are deficient in directly augmenting LEC proliferation in vitro. Additional studies demonstrated that this function of Treg cells in the setting of lung pathology can be altered by the inhibition of DNA methyltransferases or by using Treg cells from aged mice; however, the authors interpreted these findings as being due to intrinsic alterations in Treg cell function rather than effects on direct Treg cell–tissue cell interaction (Morales-Nebreda et al., 2021; Singer et al., 2015).

The study of Treg cell–epithelial cell interactions in the skin has been particularly extensive. One such study investigated the role of Treg cells in wound healing using a skin punch biopsy mouse model (Nosbaum et al., 2016). In this setting, activated Treg cells accumulate in wounded skin and Treg cell depletion prevented proper wound healing, which the authors attributed to the lack of Treg cell–mediated immunosuppression rather than direct interaction with tissue cells. Another report by this group utilized models of hair regrowth to investigate Treg cell interactions with hair follicle epithelial stem cells (HFSCs) (Ali et al., 2017). In this study, tissue-resident Treg cells of an activated phenotype were enriched during the anagen (follicle regrowth) phase of hair growth compared with the telogen (follicle quiescence) phase. Furthermore, Treg cell depletion impaired hair regrowth in a depilation model, with reduced HFSC proliferation in Treg cell–depleted mice likely being the culprit for this failure to regrow hair. Importantly, immunosuppression was not altered in this context, and similar to observations in the lung, Treg cells appear to spatially colocalize with HFSCs. The authors identified Notch ligand JAG1 as a critical Treg cell–derived mediator of HFSC proliferation, which they confirmed through the generation and experimental use of Treg cell–specific *Jag1* knockout mice.

Another set of reports by this group focused on the Treg cell–keratinocyte signaling axis. Mathur et al. (2019) used a subacute skin injury model to investigate hair follicle regeneration. In this model, Treg cells repressed CXCL5 production by keratinocytes, limiting the recruitment of inflammatory Th17 cells and neutrophils to promote healing. A later report from the same group (Moreau et al., 2021) found that Treg cell expression of αvβ8 integrin activates latent TGF-β in the surrounding tissue microenvironment, which subsequently signals to keratinocytes to induce CXCL5 production. This in turn promotes the recruitment of Th17 cells and neutrophils that inhibit regeneration. Despite the seemingly conflicting nature of these reports, the authors argue that both mechanisms are likely involved in the proper adjustment of the immune response in various contexts.

Evidence from other groups substantiates the role of Treg cell interactions with tissue cells in the skin. UVB irradiation of skin results in an increase in activated, clonally expanded Treg cells, and UVB-mediated acceleration of wound healing (using the punch biopsy model) is dependent on the presence of Treg cells (Shime et al., 2020). The authors experimentally attribute these effects to UVB-induced Treg cell expression of opioid precursor proenkephalin (Penk), a novel molecule in the tissue repair context. In another report, the authors found that skin Treg cells require glucocorticoid signaling to mediate HFSC proliferation and direct hair growth (Liu et al., 2022b). In addition, Treg cell–derived TGF-β3, induced by glucocorticoid signaling, was shown to directly engage TGF-β receptors on HFSCs to promote their activation and proliferation both in vitro and in vivo.

Although the complexity of the gastrointestinal tract’s immune system and microbiota makes studies of specific cellular interactions difficult, some recent reports have successfully elucidated Treg cell–epithelial cell relationships in this context. In one such report (Song et al., 2015), the authors explored the role of FGF2 and IL-17 in colitis models, finding that knockout of either worsens reparative intestinal epithelial cell (IEC) proliferation. Both mediators were shown to be required in tandem for proper repair, and Treg cells and Th17 cells were reported to be the likely source for each. They also found that FGF2 induction by Treg cells is microbiota and TGF-β dependent.

Despite not being considered traditional antigen-presenting cells, intestinal epithelial stem cells (ISCs) express MHC-II, making them a particularly interesting cell type to study in the context of T cell interactions. A report studying this interaction (Biton et al., 2018) demonstrated that ISCs can utilize MHC-II to stimulate antigen-specific T cell responses and that ISC-specific MHC-II deletion leads to a reduction of CD4^+^ T cells in crypts, which correlated with a profound decrease in ISC differentiation (Biton et al., 2018). The authors showed that the addition of Treg cells or provision of recombinant IL-10 could increase the number of ISCs but not differentiated cell types in vitro. These findings suggest that Treg cell–derived IL-10 may be acting directly on epithelial cells to promote stemness in a role separate from its long-appreciated immunosuppression functions.

While the brain has historically been viewed as an immune-privileged organ, it is now clear that immune cells—including Treg cells—frequently access this site, particularly during damage. In the brain, the role of Treg cells has been most extensively investigated in the model of ischemic stroke using middle cerebral artery occlusion (MCAO). There are numerous reports querying how Treg cell depletion or expansion can affect outcomes in this model, with most studies attributing the protective effects of Treg cells in ischemic stroke to their immunosuppressive capacity (Liesz et al., 2015). However, one report using the MCAO model highlighted a potential direct role of Treg cell–derived IL-10 on neuronal outgrowth (Wang et al., 2015). The authors found that activated Treg cells promoted the proliferation of a Mash1^+^ population of neural stem cells, precursors to neurons, at homeostasis and after MCAO injury in vivo, and neuron proliferation in vitro—an effect that was blocked by anti-IL-10 antibody treatment.

Addressing another neurological disease state, Dombrowski et al. (2017) investigated the role of Treg cells in remyelination of neurons using demyelination models involving lysolecithin (spinal cord) or cuprizone (corpus callosum) injury. The authors showed that oligodendrocyte progenitor cell (OPC) differentiation and subsequent remyelination are impaired upon Treg cell depletion in both models and that adoptive transfer of Treg cells can rescue proper remyelination. Furthermore, they identified matricellular growth regulatory protein CCN3 as a potential Treg cell–derived factor that stimulates OPC differentiation in several coculture models. However, a follow-up report (Dittmer et al., 2018) utilizing similar methods indicated that Treg cell–derived CCN3 may not signal directly to OPCs. Studies by a different group investigating Treg cell–induced reparative remyelination (Shi et al., 2021) found that Treg cell–derived matricellular ECM protein osteopontin signals to microglia to induce OPC differentiation, highlighting a microglia-mediated role for Treg cells in this context. However, given the multifunctional nature of ECM components, osteopontin could also impact other tissue cells in this or related disease contexts.

### Treg cell–mesenchymal cell interactions

While these earlier reports regarding Treg cell–epithelial cell interactions prompted many to view this as the primary mode of Treg cell–mediated tissue affectation, subsequent work has found that the target of Treg cell–derived signals in other tissue contexts is mesenchymal cells. Utilizing a model of lung influenza A virus (IAV) infection, an initial study investigated a potential role for Treg cell–derived production of the epidermal growth factor receptor (EGFR)–ligand amphiregulin (Areg) (Arpaia et al., 2015). Using an *Areg* conditional knockout mouse, this report showed that animals specifically lacking Areg production by Treg cells exhibited reduced blood oxygen saturation and increased lung edema during the course of infection. Notably, these conditional knockout mice displayed equivalent IAV-specific CD4^+^ and CD8^+^ T cell responses with no alterations in lung viral load, pointing to a tissue cell–driven effect on lung recovery—mediated by Treg cell–derived Areg that is separable from their canonical immunosuppressive function.

Given that epithelial cells express EGFR in many tissues/cell lines and the aforementioned findings of Treg cell–LEC interactions by other groups, it was expected that the target tissue cells of Treg cell–derived Areg were likely LECs. However, in a recent report by our group (Kaiser et al., 2023), it was found that type II alveolar epithelial cells (AT2s)—the LEC type for which activation/differentiation is necessary for lung recovery after IAV infection—express little to no *Egfr* and thus are unlikely to be direct sensors of Areg. Instead, we found that mesenchymal cells expressed the highest levels of *Egfr* in the lung and identified a particular *Egfr*-high subset of *Col14a1*-expressing lung mesenchymal cells (LMCs), which we termed Col14^*+*^ LMCs (also known as adventitial fibroblasts), that is uniquely responsive to Areg. Notably, Treg cells are spatially positioned nearby these Col14^*+*^ LMCs, and Treg cell–derived Areg was shown to specifically prevent the death of this LMC subset in IAV-infected lungs. Genetically targeting *Egfr* for deletion in mesenchymal cells further underscored that Treg cell–derived Areg signaling to LMCs mediates alveolar repair in this context. A subsequent signaling relay from LMCs to LECs likely provides support for regeneration of the latter; this is supported by studies using a Treg cell/LMC coculture model in which lung Treg cells, but not spleen Treg cells, were able to induce pronounced tissue reparative gene expression in LMCs.

Treg cell–mesenchymal cell interactions have also been highlighted in muscle tissue. In an influential early report by the Mathis/Benoist group (Burzyn et al., 2013), the authors found that Treg cells accumulate at the site of tissue damage in several models of muscle injury, and Treg cell depletion inhibited muscle repair. Finding that Treg cells from injured muscles produce large amounts of Areg, the authors determined that Treg cell–derived Areg promotes satellite cell (muscle-associated mesenchymal stem cells) proliferation and differentiation to facilitate muscle recovery. Similarly, another report (Castiglioni et al., 2015) demonstrated that Treg cells are the primary T cell type recruited to injured muscle and showed reduced muscle recovery and satellite cell mobilization after injury in *Rag2*^*–/–*^*Il2rg*^*–/–*^ mice. This report also showed that specifically Treg cells, but not other T cell populations, increase satellite cell proliferation in vitro. Other reports (Panduro et al., 2018; Villalta et al., 2014) also found detrimental effects of Treg cell depletion on repair in muscle injury models but attributed these results to increased immune activation in this context. Interestingly, in the setting of parasitic muscle infection, the presence of Treg cells may inhibit proper muscle repair due to their influence on macrophage polarization (Jin et al., 2017).

There have also been multiple reports highlighting the roles of Treg cell–mesenchymal cell interactions in cardiovascular damage. Several of these reports (Saxena et al., 2014; Sharir et al., 2014b; Tang et al., 2012; Weirather et al., 2014; Xia et al., 2020) investigate the role of heart Treg cells in myocardial infarction (MI) models, while others (Ait-Oufella et al., 2013; Li et al., 2019a; Meng et al., 2014) explore this in experimental induction of abdominal aortic aneurysm (AAA). These studies found that Treg cells accumulate in the heart and aorta during MI and AAA respectively, have a tissue-adapted phenotype disparate from Treg cells in secondary lymphoid organs, and Treg cell depletion leads to worse disease outcomes. Although the authors in these reports primarily attribute the observed pathology to heightened inflammation, some of these reports also demonstrate that beyond their immunosuppressive role, Treg cells signal to cardiomyocytes (CM) or aortic smooth muscle cells (SMCs) via IL-10 (Meng et al., 2014; Tang et al., 2012), TGF-β (Meng et al., 2014), or deposition of matricellular ECM protein SPARC (Xia et al., 2020) to mediate protection from cardiovascular disease.

Treg cell accumulation has also been observed in thrombi, with one study demonstrating that Treg cell–depletion inhibits thrombus resolution (Shahneh et al., 2021). This group also identified SPARC as a novel Treg cell–derived mediator in this context; administration of SPARC-knockout Treg cells to *Rag1*^*−/−*^ mice resulted in impaired thrombus resolution. While the authors attribute this effect to the ability of SPARC to induce monocyte MMP activity, ECM proteins are known to be pleotropic and interact with several types of tissue cells, so the effect of SPARC in this context could be multifarious.

In the setting of neonatal cryoinjury, one report investigated the role of Treg cells in heart tissue regeneration (Li et al., 2019b). Mice lacking adaptive immune cells failed to undergo proper heart regeneration, which could be rescued by adoptive transfer of Treg cells, and Treg cell depletion resulted in worsened regeneration characteristics. The authors identified three candidate Treg cell–derived mediators that promoted CM proliferation in vitro: chemokine CCL24, growth-stimulating secreted factor GAS6, and Areg. In a mouse AAA model (Li et al., 2022), another study identified a novel Treg cell–derived mediator of tissue–cell intercommunication in the context of vascular damage. This study found that Treg cells, which accumulate in injured aorta, produce trefoil factor 1 (TFF1), a lectin-like peptide component of mucous in barrier tissues. Treg cell–specific *Tff1* deletion resulted in increased damage, as measured by aortic diameter and SMC apoptosis. Correspondingly, *Tff1* overexpression improved recovery, and treatment of in vitro primary vascular SMCs with recombinant TFF1 demonstrated antiapoptotic effects.

Treg cells have also been shown to affect mesenchymal cells in the brain, where an accumulation of activated Treg cells is observed in the later stages of the MCAO model (Ito et al., 2019). In this setting, Treg cell depletion resulted in increased pathologic activation of astrocytes—brain mesenchymal cells—and impaired neurological recovery. Adoptive transfer of Treg cells improved recovery of MCAO-injured mice lacking lymphocytes. As the authors found that brain Treg cells express *Areg*, they used in vitro coculture experiments and adoptive transfer of *Areg*-knockout Treg cells to show that Treg cell–derived Areg signals to astrocytes and microglia—brain-resident macrophages—to promote the production of IL-6, which in turn ameliorates pathologic astrocyte outgrowth and improves neurologic recovery following MCAO.

Potential Treg cell–mesenchymal cell interactions have additionally been investigated in non-traditional tissue environments. One report (Camacho et al., 2020) found an enrichment of Treg cells in the mouse bone marrow (BM), demonstrating proficiency for homing to the BM following intravenous injection. The authors found that Treg cells signal to BM mesenchymal cell populations to restrict their improper growth and to support healthy hematopoietic stem cell (HSC) generation—a feature that they attributed to Treg cell–derived IL-10. Notably, disruption of this Treg cell–mesenchymal cell IL-10 axis led to deficiencies in the long-term engraftment capability of HSCs.

Further highlighting the ability of Treg cells to signal toward either epithelial cells or mesenchymal cells in different tissue contexts, a report demonstrating interspecies conservation of Treg cell–tissue cell interaction effects leveraged the high regenerative potential of zebrafish to study Treg cells in organ damage/regeneration models (Hui et al., 2017). This group identified zTreg cells, a Treg cell–like population of cells that express an ortholog of Foxp3, and showed their enrichment in regenerating the spinal cord, heart, and retina; regeneration in these tissues was profoundly dysregulated upon zTreg cell depletion. This report identified various mediators that are critical for tissue cell activation in different tissues: neuronal growth factor neurotrophin-3 (ntf3) from zTreg cells induces neural cell progenitor proliferation in damaged spinal cord, EGFR ligand neuregulin-1 (nrg1) induces CM proliferation in damaged heart, and insulin-like growth factor 1 (igf1) induces glial cell proliferation in the damaged retina. Whether these mediators are important for Treg cell–tissue cell communication in other vertebrates remains to be seen.

### Treg cell–endothelial cell interactions

As the entryway for immune cells into tissue, endothelial cells constitute an unconventional cell type often considered a subset of “tissue cells,” with several reports indicating direct interactions with Treg cells. In one such report (Maganto-García et al., 2011), the authors found that endothelial cells, via MHC-II–based antigen presentation, stimulate Treg cells to produce TGF-β, which in turn leads to changes in vascular endothelial cells that result in decreased transmigration of other T cell subsets. Fu et al. (2014) further suggested this was due to Treg cell T cell receptor (TCR)–endothelial MHC-II interactions that promote Treg cell transmigration in an allospecific manner, inhibiting the recruitment of other T cell subsets from the blood. A series of reports from the Bromberg group (Brinkman et al., 2016; Piao et al., 2020; Saxena et al., 2022) explore interactions between Treg cells and the lymphatic endothelium, showing that binding of Treg cell–derived lymphotoxin αβ to endothelial lymphotoxin receptors promotes the recruitment of Treg cells from lymphatic vessels; however, this interaction increases the recruitment of other T cell subsets.

A number of reports (Leung et al., 2018; Liu et al., 2022a; Sharir et al., 2014a; Zouggari et al., 2009) have shown a role for Treg cells in improving neovascularization in ischemic tissue models, though one report found a detrimental effect (Hellingman et al., 2012). In one such report (Leung et al., 2018), the authors found that mice with diet- or genetically induced diabetes-like states exhibited reduced reperfusion in a model of muscle ischemia. In this context, improved neovascularization was dependent on heightened Treg cell reparative activity toward endothelial cells. Further, the authors found that Treg cells, or Treg cell–conditioned media, IL-10, or Areg can directly promote endothelial cell tube formation in vitro. In a separate report, Treg cell–derived Areg was also shown to directly promote endothelial cell activity in vitro (Liu et al., 2022a).

### Treg cell “anchoring” in tissue

To potentiate their interactions with tissue cells, Treg cells have also been suggested to “anchor” themselves in tissues, in a manner that could promote optimal interaction with tissue cells. In Mock et al. (2014), the authors find that in ALI, adoptively transferred *Itgae*^*–/–*^ Treg cells are less able to engraft lung tissue than wild type Treg cells, suggesting that CD103 on Tregs may poise Treg cells for enhanced tissue cell interaction. In Kaiser et al. (2023), we made the observation that Tregs in IAV-infected lungs localize in areas deficient in certain types of extracellular matrix (ECM); Treg cell–ECM interaction has not been extensively explored, but this may be an important factor in confining Tregs to tissue regions with cognate mesenchymal cells. In the skin (Mehta et al., 2021), ECM-binding C-type lectin receptor layilin (*Layn*) was highly expressed in both mouse and human skin Treg cells. Through the generation of Treg cell–specific *Layn* knockout mice, the authors found that *Layn* is critical for Treg cell localization, with mice with Treg cell–specific *Layn* deletion having impaired wound healing.

## Activating tissue repair function in Treg cells

IL-18 and IL-33, two factors released upon tissue damage, have been shown to promote Areg production by Treg cells (Arpaia et al., 2015). IL-33 has also been demonstrated to be a critical mediator of tissue Treg cell reparative activity in the muscle (Kuswanto et al., 2016), cardiovascular system (Li et al., 2019a, 2022), and brain (Ito et al., 2019). Additional mediators reported to induce reparative Treg function include glucocorticoids in the skin (Liu et al., 2022b), serotonin in the brain (Ito et al., 2019), and commensal microbiota–derived components in the gut (Song et al., 2015). These observations suggest that damage-associated signals drive the activation of Treg cell tissue repair programs. While identifying the cell types that produce these mediators remains an active area of investigation, some reports have identified specific cellular sources in various tissue damage contexts.

Though traditionally viewed as being derived primarily from epithelial cells (Drake and Kita, 2017), a key role for IL-33 production by other tissue cell types has begun to be appreciated as important for initiating repair and regeneration. Adventitial fibroblasts are a key source of IL-33 in the lung that mediate repair following helminth infection (Dahlgren et al., 2019) and could influence Treg cell repair programs given their colocalization (Kaiser et al., 2023). Several additional examples suggest that tissue-specific fibroblast populations direct Treg cell tissue repair function. Mesenchymal cell–derived IL-33 promotes Treg cell repair programs in the damaged muscle (Kuswanto et al., 2016).

The first indication that tissue-resident Treg cells were distinct from circulating Treg cells came from studies in visceral adipose tissue (VAT) (Muñoz-Rojas and Mathis, 2021). VAT Treg cells express high levels of *Areg* (Burzyn et al., 2013) and have a close spatial interaction with IL-33–producing mesenchymal cells, with mesenchymal cell–derived IL-33 leading to the expansion of VAT Treg cells (Spallanzani et al., 2019). Further, Treg cell activity negatively regulates IL-33 expression by VAT mesenchymal cells and thus prevents aberrant VAT inflammation (Spallanzani et al., 2019). Notably, VAT Treg cells are significantly reduced in mice fed a high-fat diet as compared with mice fed normal chow (Feuerer et al., 2009; Winer et al., 2009). Adoptive transfer of Treg cells or Treg cell gain of function reduces insulin resistance in high-fat diet–fed mice, demonstrating that VAT Treg cells are critical for the maintenance of insulin sensitivity—a feature attributed to their role in immunosuppression (Feuerer et al., 2009). Together, these studies suggest that loss of VAT Treg cells in high-fat diet feeding leads to increased IL-33 levels in VAT due to unrestrained expansion of IL-33–producing mesenchymal cells, resulting in aberrant inflammation that promotes insulin resistance. However, the effect of VAT Treg cell–derived tissue repair mediators, such as Areg, on resident mesenchymal cells or adipose progenitor cells and the potential role of mesenchymal cell–derived IL-33 in driving that activity are yet to be described.

Interestingly, other tissue mediators appear to inhibit reparative Treg cell activity. For example, Harb et al. (2021) showed that Notch4 signaling in Treg cells reduces *Areg* expression and negatively impacts tissue recovery in viral infection models, indicating that inhibition of this axis could potentiate Treg cell reparative phenotypes. Global knockout of GPR174—a G-protein–coupled receptor for lysophosphatidylserine (LysoPS)—resulted in increased Treg cell generation and tissue residency (Barnes et al., 2015), while GPR174-knockout Treg cells have heightened production of Areg and increased ability to induce neovascularization in a muscle ischemia injury model (Liu et al., 2022a), suggesting LysoPS-mediated GPR174 activation inhibits Treg cell tissue reparative activity. Given the pronounced levels of LysoPS present across tissue sites (Barnes et al., 2015), further exploration of this axis with regard to Treg cell reparative phenotypes is warranted.

In the lung, Treg cells do not require expression or activation of the TCR to produce Areg (Arpaia et al., 2015); however, in the brain, TCR signaling was shown to be required (Ito et al., 2019). Based on previous work that found enrichment for a particular Treg cell TCR in the injured muscle (Burzyn et al., 2013), a follow-up study generated transgenic mice expressing this specific TCR and demonstrated that Treg cells from these animals preferentially migrate to and are retained in muscle, have a muscle repair-oriented transcriptional signature, and enhance muscle regeneration (Cho et al., 2019). However, the self-antigens that may induce this potential in muscle Tregs are yet to be identified. VAT Treg cells have also been shown to have a unique TCR repertoire (Feuerer et al., 2009; Winer et al., 2009), and transgenic TCR approaches have demonstrated a role for TCR signaling in VAT Treg cell accumulation and differentiation (Li et al., 2018); however, the role of antigen-specificity in VAT Treg cell activation or Areg production is unclear. These reports collectively suggest that the role of antigen-driven tissue repair function may be context and/or model dependent—potentially a function of the type, severity, or duration of damage in a given tissue. Although epithelial, mesenchymal, and endothelial cells are known to present antigens to Treg cells via MHC-II expression (Biton et al., 2018; Cruickshank et al., 2004; Fu et al., 2014; Maganto-García et al., 2011; Westendorf et al., 2009), whether this contributes to activation of the Treg cell tissue repair program or mediates the retention of Treg cells in specific tissues is unclear.

Overall, the studies discussed above suggest that the reparative activity of Treg cells is activated by early tissue damage–associated mediators and other niche-specific factors, with a possible additional contribution for antigen stimulation.

## Treg cell–structural cell interactions in chronic disease and cancer

### Treg cells in fibrosis and chronic tissue injury

The role of Treg cell interactions with fibroblasts and epithelial cells as pro- or anti-fibrotic in chronic tissue injury has been largely unexplored. Suggestive of their potential role in the development of tissue fibrosis, Treg cells are enriched in the livers of mice and humans with chronic liver disease (Claassen et al., 2010; Ikeno et al., 2020; Langhans et al., 2013; Savage et al., 2024). Further, in human liver cirrhosis, Treg cells are believed to interact closely with hepatic stellate cells (Langhans et al., 2013), a liver resident fibroblast population key to liver fibrosis (Mederacke et al., 2013). Treg cell depletion has also been shown to worsen liver and skin fibrosis (Ikeno et al., 2020; Kalekar et al., 2019; Katz et al., 2011), with Gata3^+^ Treg cells thought to be anti-fibrotic in mouse skin fibrosis models. However, recent work from our group demonstrated that Treg cells interact with hepatic stellate cells in multiple models of chronic liver injury, with Treg cell–derived Areg activating EGFR signaling pathways in hepatic stellate cells to promote the development of liver fibrosis (Savage et al., 2024). Further, hepatic stellate cells activated by Treg cell–derived Areg produce IL-6 that stimulates hepatocyte gluconeogenesis and leads to glucose intolerance (Savage et al., 2024).

In the lung, there is conflicting evidence suggesting Treg cells can both promote (Birjandi et al., 2016; Chakraborty et al., 2018; Xiong et al., 2015) and ameliorate lung fibrosis (Kamio et al., 2018). Using in vitro cultures and in vivo Treg cell depletion, a prior study suggested that PDGF-β from Treg cells may promote lung fibrosis by directly stimulating lung fibroblasts (Lo Re et al., 2011).

As in vivo Treg cell depletion results in increased inflammation and tissue damage that could worsen fibrosis—thereby confounding attempts to isolate the tissue repair function of Treg cells—more definitive studies of Treg cell interactions with fibroblasts in the setting of chronic disease will be required to identify critical tissue cell– and Treg cell–derived mediators that regulate fibrosis development.

### Treg cell interactions with stromal cells in cancer

Cancer is a unique chronic disease in which tumor cells often hijack normal tissue repair processes to drive growth. Across various malignancies, Treg cell–mediated immunosuppression of anti-tumor T cell responses has been extensively studied and reviewed elsewhere (Plitas and Rudensky, 2020; Togashi et al., 2019). Mechanistic studies of Treg cell–stromal interactions are a newer area of study with less extensive literature, but their importance to our understanding of cancer progression is clear and growing.

In highly desmoplastic malignancies, cancer-associated stromal cells make up a significant proportion of the tumor microenvironment (TME) and modify the immune cell landscape. Treg cells have been shown to primarily associate with stromal cells in the TME of solid tumors (Kinoshita et al., 2013), suggesting a level of crosstalk may occur between the two cell types. This is most notably studied in pancreatic ductal adenocarcinoma (PDAC)—a cancer type with a fibroblast-rich TME. In a mouse model of PDAC, depletion of Treg cells results in the functional reprogramming of cancer-associated fibroblast (CAF) populations, resulting in the loss of tumor-restraining α-SMA^+^ CAFs and tumor outgrowth (Zhang et al., 2020); the loss of Treg cell–derived TGF-β may drive α-SMA^+^ CAFs to lose their myofibroblastic phenotype, resulting in increased carcinogenesis, the generation of aberrant immune responses, and unrestricted tumor growth. Notably, this result is in contrast to what would be expected with the loss of immunosuppressive functions of Treg cells and further emphasizes the importance of studying Treg cell–CAF interactions. Furthermore, depletion of α-SMA^+^ CAFs alone stimulates tumor growth (Özdemir et al., 2014), with an increase in tumor-infiltrating Treg cells and decreased overall survival. Though Treg cells are prominent sources of TGF-β, more targeted genetic approaches are necessary to determine how Treg cell–derived TGF-β (or other mediators) specifically affect CAF function. Interestingly, in this model, Treg cells express increased levels of CTLA-4, and administration of anti-CTLA-4 antibody slowed PDAC progression and increased overall survival.

Beyond TGF-β, Treg cell–derived Areg drives greater lung tumor burden in a model of metastatic breast cancer (Green et al., 2017), although the target cells through which Areg mediates this effect are unknown. It is possible that Treg cell–derived Areg signals through stromal cells in lung cancer, as it does in other disease contexts discussed above (Kaiser et al., 2023), to promote tumor growth. To understand how Treg cells modulate CAF phenotypes to potentially regulate carcinogenesis, further investigation of Treg cell–CAF interactions, including the identification of other mediators, will be required.

Crosstalk between CAFs and Treg cells is bidirectional, with CAF populations influencing Treg cell functional dynamics in the TME. Interestingly, fibroblast populations with the capacity to present antigen (apCAFs) have been shown to promote the induction of Foxp3^+^ Treg cells from naïve CD4^+^ T cells (Huang et al., 2022). Moreover, these Treg cells suppress antigen-specific T cell responses. Though apCAF populations have been shown to impact antigen-specific CD4^+^ T cell responses in several models of cancer (Kerdidani et al., 2022), the overall impact of apCAF-induced Treg cells on cancer outcomes remains largely understudied. More broadly, studies have demonstrated that fibroblast-derived chemokines such as CCL5 and CXCL12 lead to the specific migration and retention of Treg cells to tumors (Cheng et al., 2018; Costa et al., 2018; Givel et al., 2018; Karnoub et al., 2007), which can influence the level of immunosuppression in the TME and overall survival.

Treg cell–derived factors have also been implicated in promoting endothelial sprouting in cancer. Facciabene et al. (2011) demonstrated that tumor hypoxia leads to the recruitment of CCR10^+^ Treg cells, which secrete high levels of VEGF, and ablation of these Treg cells suppressed angiogenesis. Moreover, increased angiogenic sprouting was observed in in vitro assays utilizing hypoxic Treg cell–conditioned medium.

**Figure 3.**
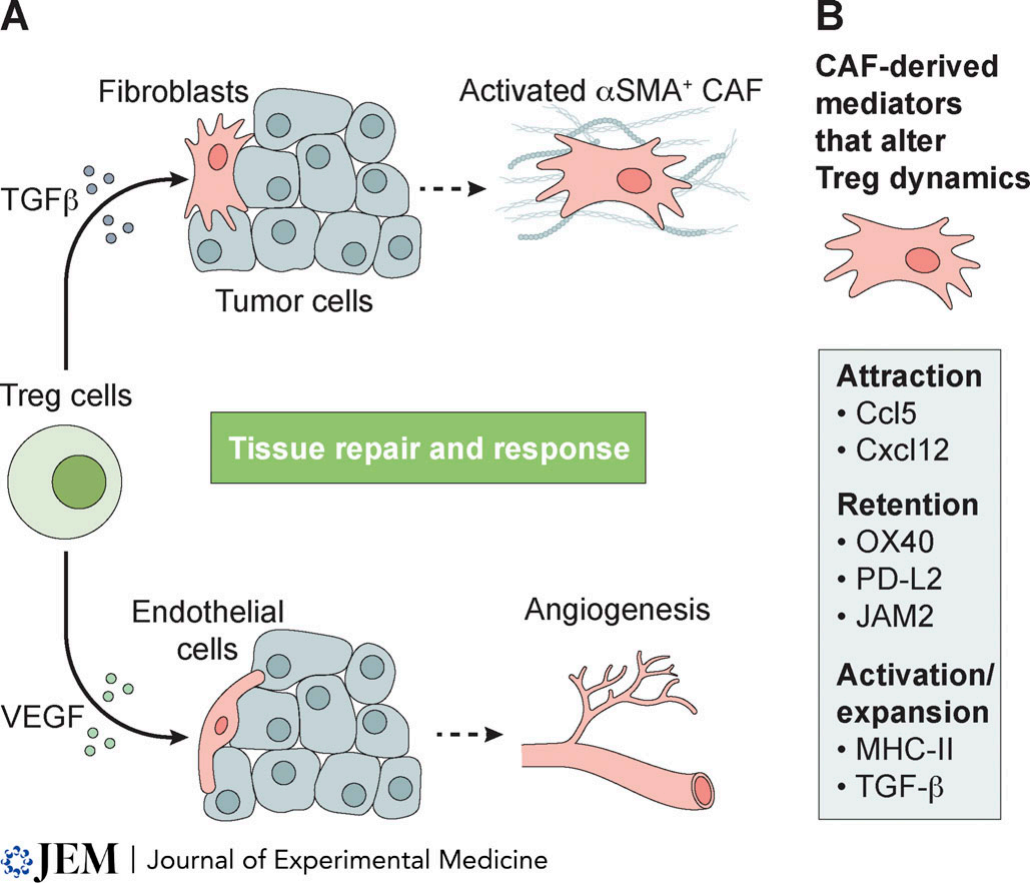
**Treg cell repair function in cancer. (A)** Treg cell–derived mediators are sensed by cancer-associated stromal cells and influence their function. **(B)** Specific CAF–Treg cell interactions can influence Treg cell phenotype and function in the TME.

The activation of tissue repair functionality in tumor-associated Treg cells may drive a tissue-specific, wound-healing modality of cancer-associated stromal cells (Fig. 3). The effect of this interaction is that highly context-dependent—tissue-specific programs likely guide the function and phenotype of tumor-associated stromal cells—thus, continued mechanistic studies of Treg cell–stromal interactions in cancer will be needed in order fill this knowledge gap.

## Concluding remarks

In this review, we have discussed the roles of Treg cells in promoting tissue repair/regeneration through interactions with tissue-resident parenchymal and structural cells, a non-canonical function that is distinct from their immunosuppressive properties. We have highlighted numerous studies examining Treg cell–derived mediators that underpin these interactions and their importance for tissue repair and regeneration—recognizing the critical role tissue cells play in translating common Treg cell–derived inputs into tissue-specific outputs. These processes suggest potential targets for the treatment of human disease; however, there remains much to learn in terms of identifying additional factors and their associated mechanisms of action, especially in the setting of chronic disease. Overall, the interplay of Treg cells with non-immune cells offers an exciting new area of study in the field of Treg cell biology that could further our understanding of mechanisms that maintain tissue integrity.

## References

[bib1] Ait-Oufella, H., Y. Wang, O. Herbin, S. Bourcier, S. Potteaux, J. Joffre, X. Loyer, P. Ponnuswamy, B. Esposito, M. Dalloz, . 2013. Natural regulatory T cells limit angiotensin II-induced aneurysm formation and rupture in mice. Arterioscler. Thromb. Vasc. Biol. 33:2374–2379. 10.1161/ATVBAHA.113.301280 23908246

[bib2] Ali, N., B. Zirak, R.S. Rodriguez, M.L. Pauli, H.A. Truong, K. Lai, R. Ahn, K. Corbin, M.M. Lowe, T.C. Scharschmidt, . 2017. Regulatory T cells in skin facilitate epithelial stem cell differentiation. Cell. 169:1119–1129.e11. 10.1016/j.cell.2017.05.002 28552347 PMC5504703

[bib3] Arpaia, N., J.A. Green, B. Moltedo, A. Arvey, S. Hemmers, S. Yuan, P.M. Treuting, and A.Y. Rudensky. 2015. A distinct function of regulatory T cells in tissue protection. Cell. 162:1078–1089. 10.1016/j.cell.2015.08.021 26317471 PMC4603556

[bib4] Astarita, J.L., C.X. Dominguez, C. Tan, J. Guillen, M.L. Pauli, R. Labastida, J. Valle, M. Kleinschek, J. Lyons, and A.A. Zarrin. 2023. Treg specialization and functions beyond immune suppression. Clin. Exp. Immunol. 211:176–183. 10.1093/cei/uxac123 36571811 PMC10019124

[bib5] Barnes, M.J., C.M. Li, Y. Xu, J. An, Y. Huang, and J.G. Cyster. 2015. The lysophosphatidylserine receptor GPR174 constrains regulatory T cell development and function. J. Exp. Med. 212:1011–1020. 10.1084/jem.20141827 26077720 PMC4493414

[bib6] Birjandi, S.Z., V. Palchevskiy, Y.Y. Xue, S. Nunez, R. Kern, S.S. Weigt, J.P. Lynch III, T.A. Chatila, and J.A. Belperio. 2016. CD4(+)CD25(hi)Foxp3(+) cells exacerbate bleomycin-induced pulmonary fibrosis. Am. J. Pathol. 186:2008–2020. 10.1016/j.ajpath.2016.03.020 27317904 PMC4973661

[bib7] Biton, M., A.L. Haber, N. Rogel, G. Burgin, S. Beyaz, A. Schnell, O. Ashenberg, C.W. Su, C. Smillie, K. Shekhar, . 2018. T helper cell cytokines modulate intestinal stem cell renewal and differentiation. Cell. 175:1307–1320.e22. 10.1016/j.cell.2018.10.008 30392957 PMC6239889

[bib8] Boothby, I.C., J.N. Cohen, and M.D. Rosenblum. 2020. Regulatory T cells in skin injury: At the crossroads of tolerance and tissue repair. Sci Immunol. 5:eaaz9631. 10.1126/sciimmunol.aaz9631 32358172 PMC7274208

[bib9] Brinkman, C.C., D. Iwami, M.K. Hritzo, Y. Xiong, S. Ahmad, T. Simon, K.L. Hippen, B.R. Blazar, and J.S. Bromberg. 2016. Treg engage lymphotoxin beta receptor for afferent lymphatic transendothelial migration. Nat. Commun. 7:12021. 10.1038/ncomms12021 27323847 PMC4919545

[bib10] Burzyn, D., W. Kuswanto, D. Kolodin, J.L. Shadrach, M. Cerletti, Y. Jang, E. Sefik, T.G. Tan, A.J. Wagers, C. Benoist, and D. Mathis. 2013. A special population of regulatory T cells potentiates muscle repair. Cell. 155:1282–1295. 10.1016/j.cell.2013.10.054 24315098 PMC3894749

[bib11] Camacho, V., V.R. Matkins, S.B. Patel, J.M. Lever, Z. Yang, L. Ying, A.E. Landuyt, E.C. Dean, J.F. George, H. Yang, . 2020. Bone marrow Tregs mediate stromal cell function and support hematopoiesis via IL-10. JCI Insight. 5:e135681. 10.1172/jci.insight.135681 33208555 PMC7710301

[bib12] Castiglioni, A., G. Corna, E. Rigamonti, V. Basso, M. Vezzoli, A. Monno, A.E. Almada, A. Mondino, A.J. Wagers, A.A. Manfredi, and P. Rovere-Querini. 2015. FOXP3+ T cells recruited to sites of sterile skeletal muscle injury regulate the fate of satellite cells and guide effective tissue regeneration. PLoS One. 10:e0128094. 10.1371/journal.pone.0128094 26039259 PMC4454513

[bib13] Chakraborty, K., S. Chatterjee, and A. Bhattacharyya. 2018. Impact of Treg on other T cell subsets in progression of fibrosis in experimental lung fibrosis. Tissue Cell. 53:87–92. 10.1016/j.tice.2018.06.003 30060832

[bib14] Cheng, H.W., L. Onder, J. Cupovic, M. Boesch, M. Novkovic, N. Pikor, I. Tarantino, R. Rodriguez, T. Schneider, W. Jochum, . 2018. CCL19-producing fibroblastic stromal cells restrain lung carcinoma growth by promoting local antitumor T-cell responses. J. Allergy Clin. Immunol. 142:1257–1271.e4. 10.1016/j.jaci.2017.12.998 29391257

[bib15] Cho, J., W. Kuswanto, C. Benoist, and D. Mathis. 2019. T cell receptor specificity drives accumulation of a reparative population of regulatory T cells within acutely injured skeletal muscle. Proc. Natl. Acad. Sci. USA. 116:26727–26733. 10.1073/pnas.1914848116 31822623 PMC6936428

[bib16] Claassen, M.A., R.J. de Knegt, H.W. Tilanus, H.L. Janssen, and A. Boonstra. 2010. Abundant numbers of regulatory T cells localize to the liver of chronic hepatitis C infected patients and limit the extent of fibrosis. J. Hepatol. 52:315–321. 10.1016/j.jhep.2009.12.013 20129690

[bib17] Costa, A., Y. Kieffer, A. Scholer-Dahirel, F. Pelon, B. Bourachot, M. Cardon, P. Sirven, I. Magagna, L. Fuhrmann, C. Bernard, . 2018. Fibroblast heterogeneity and immunosuppressive environment in human breast cancer. Cancer Cell. 33:463–479.e10. 10.1016/j.ccell.2018.01.011 29455927

[bib18] Cruickshank, S.M., L.D. McVay, D.C. Baumgart, P.J. Felsburg, and S.R. Carding. 2004. Colonic epithelial cell mediated suppression of CD4 T cell activation. Gut. 53:678–684. 10.1136/gut.2003.029967 15082586 PMC1774036

[bib19] D’Alessio, F.R., K. Tsushima, N.R. Aggarwal, E.E. West, M.H. Willett, M.F. Britos, M.R. Pipeling, R.G. Brower, R.M. Tuder, J.F. McDyer, and L.S. King. 2009. CD4+CD25+Foxp3+ Tregs resolve experimental lung injury in mice and are present in humans with acute lung injury. J. Clin. Invest. 119:2898–2913. 10.1172/JCI36498 19770521 PMC2752062

[bib20] Dahlgren, M.W., S.W. Jones, K.M. Cautivo, A. Dubinin, J.F. Ortiz-Carpena, S. Farhat, K.S. Yu, K. Lee, C. Wang, A.V. Molofsky, . 2019. Adventitial stromal cells define group 2 innate lymphoid cell tissue niches. Immunity. 50:707–722.e6. 10.1016/j.immuni.2019.02.002 30824323 PMC6553479

[bib21] Dial, C.F., M.K. Tune, C.M. Doerschuk, and J.R. Mock. 2017. Foxp3^+^ regulatory T cell expression of keratinocyte growth factor enhances lung epithelial proliferation. Am. J. Respir. Cell Mol. Biol. 57:162–173. 10.1165/rcmb.2017-0019OC 28296468 PMC5576587

[bib22] Dikiy, S., and A.Y. Rudensky. 2023. Principles of regulatory T cell function. Immunity. 56:240–255. 10.1016/j.immuni.2023.01.004 36792571

[bib23] Dittmer, M., A. Young, T. O’Hagan, G. Eleftheriadis, P. Bankhead, Y. Dombrowski, R.J. Medina, and D.C. Fitzgerald. 2018. Characterization of a murine mixed neuron-glia model and cellular responses to regulatory T cell-derived factors. Mol. Brain. 11:25. 10.1186/s13041-018-0367-6 29720228 PMC5932845

[bib24] Dombrowski, Y., T. O’Hagan, M. Dittmer, R. Penalva, S.R. Mayoral, P. Bankhead, S. Fleville, G. Eleftheriadis, C. Zhao, M. Naughton, . 2017. Regulatory T cells promote myelin regeneration in the central nervous system. Nat. Neurosci. 20:674–680. 10.1038/nn.4528 28288125 PMC5409501

[bib25] Drake, L.Y., and H. Kita. 2017. IL-33: Biological properties, functions, and roles in airway disease. Immunol. Rev. 278:173–184. 10.1111/imr.12552 28658560 PMC5492954

[bib26] Estrada Brull, A., C. Panetti, and N. Joller. 2022. Moving to the outskirts: Interplay between regulatory T cells and peripheral tissues. Front. Immunol. 13:864628. 10.3389/fimmu.2022.864628 35572535 PMC9099010

[bib27] Facciabene, A., X. Peng, I.S. Hagemann, K. Balint, A. Barchetti, L.P. Wang, P.A. Gimotty, C.B. Gilks, P. Lal, L. Zhang, and G. Coukos. 2011. Tumour hypoxia promotes tolerance and angiogenesis via CCL28 and T(reg) cells. Nature. 475:226–230. 10.1038/nature10169 21753853

[bib28] Feuerer, M., L. Herrero, D. Cipolletta, A. Naaz, J. Wong, A. Nayer, J. Lee, A.B. Goldfine, C. Benoist, S. Shoelson, and D. Mathis. 2009. Lean, but not obese, fat is enriched for a unique population of regulatory T cells that affect metabolic parameters. Nat. Med. 15:930–939. 10.1038/nm.2002 19633656 PMC3115752

[bib29] Fontenot, J.D., M.A. Gavin, and A.Y. Rudensky. 2003. Foxp3 programs the development and function of CD4+CD25+ regulatory T cells. Nat. Immunol. 4:330–336. 10.1038/ni904 12612578

[bib30] Fu, H., M. Kishore, B. Gittens, G. Wang, D. Coe, I. Komarowska, E. Infante, A.J. Ridley, D. Cooper, M. Perretti, and F.M. Marelli-Berg. 2014. Self-recognition of the endothelium enables regulatory T-cell trafficking and defines the kinetics of immune regulation. Nat. Commun. 5:3436. 10.1038/ncomms4436 24625653 PMC3959214

[bib31] Fujisaki, J., J. Wu, A.L. Carlson, L. Silberstein, P. Putheti, R. Larocca, W. Gao, T.I. Saito, C. Lo Celso, H. Tsuyuzaki, . 2011. In vivo imaging of Treg cells providing immune privilege to the haematopoietic stem-cell niche. Nature. 474:216–219. 10.1038/nature10160 21654805 PMC3725645

[bib32] Garibaldi, B.T., F.R. D’Alessio, J.R. Mock, D.C. Files, E. Chau, Y. Eto, M.B. Drummond, N.R. Aggarwal, V. Sidhaye, and L.S. King. 2013. Regulatory T cells reduce acute lung injury fibroproliferation by decreasing fibrocyte recruitment. Am. J. Respir. Cell Mol. Biol. 48:35–43. 10.1165/rcmb.2012-0198OC 23002097 PMC3547087

[bib33] Givel, A.M., Y. Kieffer, A. Scholer-Dahirel, P. Sirven, M. Cardon, F. Pelon, I. Magagna, G. Gentric, A. Costa, C. Bonneau, . 2018. miR200-regulated CXCL12β promotes fibroblast heterogeneity and immunosuppression in ovarian cancers. Nat. Commun. 9:1056. 10.1038/s41467-018-03348-z 29535360 PMC5849633

[bib34] Green, J.A., N. Arpaia, M. Schizas, A. Dobrin, and A.Y. Rudensky. 2017. A nonimmune function of T cells in promoting lung tumor progression. J. Exp. Med. 214:3565–3575. 10.1084/jem.20170356 29038367 PMC5716034

[bib35] Harb, H., M. Benamar, P.S. Lai, P. Contini, J.W. Griffith, E. Crestani, K. Schmitz-Abe, Q. Chen, J. Fong, L. Marri, . 2021. Notch4 signaling limits regulatory T-cell-mediated tissue repair and promotes severe lung inflammation in viral infections. Immunity. 54:1186–1199.e7. 10.1016/j.immuni.2021.04.002 33915108 PMC8080416

[bib36] Hellingman, A.A., L.E. van der Vlugt, M.A. Lijkwan, A.J. Bastiaansen, T. Sparwasser, H.H. Smits, J.F. Hamming, and P.H. Quax. 2012. A limited role for regulatory T cells in post-ischemic neovascularization. J. Cell. Mol. Med. 16:328–336. 10.1111/j.1582-4934.2011.01300.x 21426486 PMC3823296

[bib37] Hori, S., T. Nomura, and S. Sakaguchi. 2003. Control of regulatory T cell development by the transcription factor Foxp3. Science. 299:1057–1061. 10.1126/science.1079490 12522256

[bib38] Huang, H., Z. Wang, Y. Zhang, R.N. Pradhan, D. Ganguly, R. Chandra, G. Murimwa, S. Wright, X. Gu, R. Maddipati, . 2022. Mesothelial cell-derived antigen-presenting cancer-associated fibroblasts induce expansion of regulatory T cells in pancreatic cancer. Cancer Cell. 40:656–673.e7. 10.1016/j.ccell.2022.04.011 35523176 PMC9197998

[bib39] Hui, S.P., D.Z. Sheng, K. Sugimoto, A. Gonzalez-Rajal, S. Nakagawa, D. Hesselson, and K. Kikuchi. 2017. Zebrafish regulatory T cells mediate organ-specific regenerative programs. Dev. Cell. 43:659–672.e5. 10.1016/j.devcel.2017.11.010 29257949

[bib40] Ikeno, Y., D. Ohara, Y. Takeuchi, H. Watanabe, G. Kondoh, K. Taura, S. Uemoto, and K. Hirota. 2020. Foxp3+ regulatory T cells inhibit CCl4-induced liver inflammation and fibrosis by regulating tissue cellular immunity. Front. Immunol. 11:584048. 10.3389/fimmu.2020.584048 33178216 PMC7593684

[bib41] Ito, M., K. Komai, S. Mise-Omata, M. Iizuka-Koga, Y. Noguchi, T. Kondo, R. Sakai, K. Matsuo, T. Nakayama, O. Yoshie, . 2019. Brain regulatory T cells suppress astrogliosis and potentiate neurological recovery. Nature. 565:246–250. 10.1038/s41586-018-0824-5 30602786

[bib42] Jin, R.M., S.J. Blair, J. Warunek, R.R. Heffner, I.J. Blader, and E.A. Wohlfert. 2017. Regulatory T cells promote myositis and muscle damage in toxoplasma gondii infection. J. Immunol. 198:352–362. 10.4049/jimmunol.1600914 27895180 PMC5173414

[bib43] Josefowicz, S.Z., L.F. Lu, and A.Y. Rudensky. 2012. Regulatory T cells: Mechanisms of differentiation and function. Annu. Rev. Immunol. 30:531–564. 10.1146/annurev.immunol.25.022106.141623 22224781 PMC6066374

[bib44] Jovisic, M., N. Mambetsariev, B.D. Singer, and L. Morales-Nebreda. 2023. Differential roles of regulatory T cells in acute respiratory infections. J. Clin. Invest. 133:e170505. 10.1172/JCI170505 37463441 PMC10348770

[bib45] Kaiser, K.A., L.F. Loffredo, K.L. Santos-Alexis, O.R. Ringham, and N. Arpaia. 2023. Regulation of the alveolar regenerative niche by amphiregulin-producing regulatory T cells. J. Exp. Med. 220:e20221462. 10.1084/jem.20221462 36534084 PMC9767680

[bib46] Kalekar, L.A., J.N. Cohen, N. Prevel, P.M. Sandoval, A.N. Mathur, J.M. Moreau, M.M. Lowe, A. Nosbaum, P.J. Wolters, A. Haemel, . 2019. Regulatory T cells in skin are uniquely poised to suppress profibrotic immune responses. Sci. Immunol. 4:eaaw2910. 10.1126/sciimmunol.aaw2910 31492709 PMC6848056

[bib47] Kamio, K., A. Azuma, K. Matsuda, J. Usuki, M. Inomata, A. Morinaga, T. Kashiwada, N. Nishijima, S. Itakura, N. Kokuho, . 2018. Resolution of bleomycin-induced murine pulmonary fibrosis via a splenic lymphocyte subpopulation. Respir. Res. 19:71. 10.1186/s12931-018-0783-2 29690905 PMC5978999

[bib48] Karnoub, A.E., A.B. Dash, A.P. Vo, A. Sullivan, M.W. Brooks, G.W. Bell, A.L. Richardson, K. Polyak, R. Tubo, and R.A. Weinberg. 2007. Mesenchymal stem cells within tumour stroma promote breast cancer metastasis. Nature. 449:557–563. 10.1038/nature06188 17914389

[bib49] Katz, S.C., K. Ryan, N. Ahmed, G. Plitas, U.I. Chaudhry, T.P. Kingham, S. Naheed, C. Nguyen, P. Somasundar, N.J. Espat, . 2011. Obstructive jaundice expands intrahepatic regulatory T cells, which impair liver T lymphocyte function but modulate liver cholestasis and fibrosis. J. Immunol. 187:1150–1156. 10.4049/jimmunol.1004077 21697460 PMC3372324

[bib50] Kerdidani, D., E. Aerakis, K.M. Verrou, I. Angelidis, K. Douka, M.A. Maniou, P. Stamoulis, K. Goudevenou, A. Prados, C. Tzaferis, . 2022. Lung tumor MHCII immunity depends on in situ antigen presentation by fibroblasts. J. Exp. Med. 219:e20210815. 10.1084/jem.20210815 35029648 PMC8764966

[bib51] Kinoshita, T., G. Ishii, N. Hiraoka, S. Hirayama, C. Yamauchi, K. Aokage, T. Hishida, J. Yoshida, K. Nagai, and A. Ochiai. 2013. Forkhead box P3 regulatory T cells coexisting with cancer associated fibroblasts are correlated with a poor outcome in lung adenocarcinoma. Cancer Sci. 104:409–415. 10.1111/cas.12099 23305175 PMC7657221

[bib52] Kuswanto, W., D. Burzyn, M. Panduro, K.K. Wang, Y.C. Jang, A.J. Wagers, C. Benoist, and D. Mathis. 2016. Poor repair of skeletal muscle in aging mice reflects a defect in local, interleukin-33-dependent accumulation of regulatory T cells. Immunity. 44:355–367. 10.1016/j.immuni.2016.01.009 26872699 PMC4764071

[bib53] Langhans, B., B. Krämer, M. Louis, H.D. Nischalke, R. Hüneburg, A. Staratschek-Jox, M. Odenthal, S. Manekeller, M. Schepke, J. Kalff, . 2013. Intrahepatic IL-8 producing Foxp3⁺CD4⁺ regulatory T cells and fibrogenesis in chronic hepatitis C. J. Hepatol. 59:229–235. 10.1016/j.jhep.2013.04.011 23624000

[bib54] Leung, O.M., J. Li, X. Li, V.W. Chan, K.Y. Yang, M. Ku, L. Ji, H. Sun, H. Waldmann, X.Y. Tian, . 2018. Regulatory T cells promote apelin-mediated sprouting angiogenesis in type 2 diabetes. Cell Rep. 24:1610–1626. 10.1016/j.celrep.2018.07.019 30089270

[bib55] Li, C., J.R. DiSpirito, D. Zemmour, R.G. Spallanzani, W. Kuswanto, C. Benoist, and D. Mathis. 2018. TCR transgenic mice reveal stepwise, multi-site acquisition of the distinctive fat-treg phenotype. Cell. 174:285–299.e12. 10.1016/j.cell.2018.05.004 29887374 PMC6046274

[bib56] Li, J., N. Xia, D. Li, S. Wen, S. Qian, Y. Lu, M. Gu, T. Tang, J. Jiao, B. Lv, . 2022. Aorta regulatory T cells with a tissue-specific phenotype and function promote tissue repair through Tff1 in abdominal aortic aneurysms. Adv. Sci. 9:e2104338. 10.1002/advs.202104338 PMC8948580 35332699

[bib57] Li, J., N. Xia, S. Wen, D. Li, Y. Lu, M. Gu, T. Tang, J. Jiao, B. Lv, S. Nie, . 2019a. IL (Interleukin)-33 suppresses abdominal aortic aneurysm by enhancing regulatory T-cell expansion and activity. Arterioscler. Thromb. Vasc. Biol. 39:446–458. 10.1161/ATVBAHA.118.312023 30651000 PMC6393188

[bib58] Li, J., K.Y. Yang, R.C.Y. Tam, V.W. Chan, H.Y. Lan, S. Hori, B. Zhou, and K.O. Lui. 2019b. Regulatory T-cells regulate neonatal heart regeneration by potentiating cardiomyocyte proliferation in a paracrine manner. Theranostics. 9:4324–4341. 10.7150/thno.32734 31285764 PMC6599663

[bib59] Liesz, A., X. Hu, C. Kleinschnitz, and H. Offner. 2015. Functional role of regulatory lymphocytes in stroke: Facts and controversies. Stroke. 46:1422–1430. 10.1161/STROKEAHA.114.008608 25791715 PMC4414876

[bib60] Liu, J., L. Pan, W. Hong, S. Chen, P. Bai, W. Luo, X. Sun, F. He, X. Jia, J. Cai, . 2022a. GPR174 knockdown enhances blood flow recovery in hindlimb ischemia mice model by upregulating AREG expression. Nat. Commun. 13:7519. 10.1038/s41467-022-35159-8 36473866 PMC9727025

[bib61] Liu, Z., X. Hu, Y. Liang, J. Yu, H. Li, M.N. Shokhirev, and Y. Zheng. 2022b. Glucocorticoid signaling and regulatory T cells cooperate to maintain the hair-follicle stem-cell niche. Nat. Immunol. 23:1086–1097. 10.1038/s41590-022-01244-9 35739197 PMC9283297

[bib62] Lo Re, S., M. Lecocq, F. Uwambayinema, Y. Yakoub, M. Delos, J.B. Demoulin, S. Lucas, T. Sparwasser, J.C. Renauld, D. Lison, and F. Huaux. 2011. Platelet-derived growth factor-producing CD4+ Foxp3+ regulatory T lymphocytes promote lung fibrosis. Am. J. Respir. Crit. Care Med. 184:1270–1281. 10.1164/rccm.201103-0516OC 21868503

[bib63] Maganto-García, E., D.X. Bu, M.L. Tarrio, P. Alcaide, G. Newton, G.K. Griffin, K.J. Croce, F.W. Luscinskas, A.H. Lichtman, and N. Grabie. 2011. Foxp3+-inducible regulatory T cells suppress endothelial activation and leukocyte recruitment. J. Immunol. 187:3521–3529. 10.4049/jimmunol.1003947 21873519 PMC3217244

[bib64] Mathur, A.N., B. Zirak, I.C. Boothby, M. Tan, J.N. Cohen, T.M. Mauro, P. Mehta, M.M. Lowe, A.K. Abbas, N. Ali, and M.D. Rosenblum. 2019. Treg-cell control of a CXCL5-IL-17 inflammatory Axis promotes hair-follicle-stem-cell differentiation during skin-barrier repair. Immunity. 50:655–667.e4. 10.1016/j.immuni.2019.02.013 30893588 PMC6507428

[bib65] Mederacke, I., C.C. Hsu, J.S. Troeger, P. Huebener, X. Mu, D.H. Dapito, J.P. Pradere, and R.F. Schwabe. 2013. Fate tracing reveals hepatic stellate cells as dominant contributors to liver fibrosis independent of its aetiology. Nat. Commun. 4:2823. 10.1038/ncomms3823 24264436 PMC4059406

[bib66] Mehta, P., V. Gouirand, D.P. Boda, J. Zhang, S.V. Gearty, B. Zirak, M.M. Lowe, S. Clancy, I. Boothby, K.M. Mahuron, . 2021. Layilin anchors regulatory T cells in skin. J. Immunol. 207:1763–1775. 10.4049/jimmunol.2000970 34470859 PMC8489406

[bib67] Meng, X., J. Yang, K. Zhang, G. An, J. Kong, F. Jiang, Y. Zhang, and C. Zhang. 2014. Regulatory T cells prevent angiotensin II-induced abdominal aortic aneurysm in apolipoprotein E knockout mice. Hypertension. 64:875–882. 10.1161/HYPERTENSIONAHA.114.03950 25024283

[bib68] Mock, J.R., B.T. Garibaldi, N.R. Aggarwal, J. Jenkins, N. Limjunyawong, B.D. Singer, E. Chau, R. Rabold, D.C. Files, V. Sidhaye, . 2014. Foxp3+ regulatory T cells promote lung epithelial proliferation. Mucosal Immunol. 7:1440–1451. 10.1038/mi.2014.33 24850425 PMC4205163

[bib69] Morales-Nebreda, L., K.A. Helmin, M.A. Torres Acosta, N.S. Markov, J.Y. Hu, A.M. Joudi, R. Piseaux-Aillon, H. Abdala-Valencia, Y. Politanska, and B.D. Singer. 2021. Aging imparts cell-autonomous dysfunction to regulatory T cells during recovery from influenza pneumonia. Sci. Immunol. 6:e141690. 10.1126/sciimmunol.abg2329 PMC8026188 33600379

[bib70] Moreau, J.M., M.O. Dhariwala, V. Gouirand, D.P. Boda, I.C. Boothby, M.M. Lowe, J.N. Cohen, C.E. Macon, J.M. Leech, L.A. Kalekar, . 2021. Regulatory T cells promote innate inflammation after skin barrier breach via TGF-β activation. Sci. Immunol. 6:eabg2329. 10.1126/sciimmunol.abg2329 34452925 PMC8958044

[bib71] Muñoz-Rojas, A.R., and D. Mathis. 2021. Tissue regulatory T cells: Regulatory chameleons. Nat. Rev. Immunol. 21:597–611. 10.1038/s41577-021-00519-w 33772242 PMC8403160

[bib72] Nosbaum, A., N. Prevel, H.A. Truong, P. Mehta, M. Ettinger, T.C. Scharschmidt, N.H. Ali, M.L. Pauli, A.K. Abbas, and M.D. Rosenblum. 2016. Cutting edge: Regulatory T cells facilitate cutaneous wound healing. J. Immunol. 196:2010–2014. 10.4049/jimmunol.1502139 26826250 PMC4761457

[bib73] Özdemir, B.C., T. Pentcheva-Hoang, J.L. Carstens, X. Zheng, C.C. Wu, T.R. Simpson, H. Laklai, H. Sugimoto, C. Kahlert, S.V. Novitskiy, . 2014. Depletion of carcinoma-associated fibroblasts and fibrosis induces immunosuppression and accelerates pancreas cancer with reduced survival. Cancer Cell. 25:719–734. 10.1016/j.ccr.2014.04.005 24856586 PMC4180632

[bib74] Panduro, M., C. Benoist, and D. Mathis. 2016. Tissue Tregs. Annu. Rev. Immunol. 34:609–633. 10.1146/annurev-immunol-032712-095948 27168246 PMC4942112

[bib75] Panduro, M., C. Benoist, and D. Mathis. 2018. T_reg_ cells limit IFN-γ production to control macrophage accrual and phenotype during skeletal muscle regeneration. Proc. Natl. Acad. Sci. USA. 115:E2585–E2593. 10.1073/pnas.1800618115 29476012 PMC5856564

[bib76] Piao, W., Y. Xiong, L. Li, V. Saxena, K.D. Smith, K.L. Hippen, C. Paluskievicz, M. Willsonshirkey, B.R. Blazar, R. Abdi, and J.S. Bromberg. 2020. Regulatory T cells condition lymphatic endothelia for enhanced transendothelial migration. Cell Rep. 30:1052–1062.e5. 10.1016/j.celrep.2019.12.083 31995749 PMC7009789

[bib77] Plitas, G., and A.Y. Rudensky. 2020. Regulatory T cells in cancer. Annu. Rev. Cancer Biol. 4:459–477. 10.1146/annurev-cancerbio-030419-033428

[bib78] Sakaguchi, S., N. Mikami, J.B. Wing, A. Tanaka, K. Ichiyama, and N. Ohkura. 2020. Regulatory T cells and human disease. Annu. Rev. Immunol. 38:541–566. 10.1146/annurev-immunol-042718-041717 32017635

[bib79] Savage, T.M., K.T. Fortson, K. de Los Santos-Alexis, A. Oliveras-Alsina, M. Rouanne, S.S. Rae, J.R. Gamarra, H. Shayya, A. Kornberg, R. Cavero, . 2024. Amphiregulin from regulatory T cells promotes liver fibrosis and insulin resistance in non-alcoholic steatohepatitis. Immunity. 57:303–318.e6. 10.1016/j.immuni.2024.01.009 38309273 PMC10922825

[bib80] Saxena, A., M. Dobaczewski, V. Rai, Z. Haque, W. Chen, N. Li, and N.G. Frangogiannis. 2014. Regulatory T cells are recruited in the infarcted mouse myocardium and may modulate fibroblast phenotype and function. Am. J. Physiol. Heart Circ. Physiol. 307:H1233–H1242. 10.1152/ajpheart.00328.2014 25128167 PMC4200341

[bib81] Saxena, V., W. Piao, L. Li, C. Paluskievicz, Y. Xiong, T. Simon, R. Lakhan, C.C. Brinkman, S. Walden, K.L. Hippen, . 2022. Treg tissue stability depends on lymphotoxin beta-receptor- and adenosine-receptor-driven lymphatic endothelial cell responses. Cell Rep. 39:110727. 10.1016/j.celrep.2022.110727 35443187 PMC9093052

[bib82] Shahneh, F., A. Grill, M. Klein, F. Frauhammer, T. Bopp, K. Schäfer, V.K. Raker, and C. Becker. 2021. Specialized regulatory T cells control venous blood clot resolution through SPARC. Blood. 137:1517–1526. 10.1182/blood.2020005407 32932520

[bib83] Sharir, R., J. Semo, A. Shaish, N. Landa-Rouben, M. Entin-Meer, G. Keren, and J. George. 2014a. Regulatory T cells influence blood flow recovery in experimental hindlimb ischaemia in an IL-10-dependent manner. Cardiovasc. Res. 103:585–596. 10.1093/cvr/cvu159 24966183

[bib84] Sharir, R., J. Semo, S. Shimoni, T. Ben-Mordechai, N. Landa-Rouben, S. Maysel-Auslender, A. Shaish, M. Entin-Meer, G. Keren, and J. George. 2014b. Experimental myocardial infarction induces altered regulatory T cell hemostasis, and adoptive transfer attenuates subsequent remodeling. PLoS One. 9:e113653. 10.1371/journal.pone.0113653 25436994 PMC4249913

[bib85] Shi, L., Z. Sun, W. Su, F. Xu, D. Xie, Q. Zhang, X. Dai, K. Iyer, T.K. Hitchens, L.M. Foley, . 2021. Treg cell-derived osteopontin promotes microglia-mediated white matter repair after ischemic stroke. Immunity. 54:1527–1542.e1528. 10.1016/j.immuni.2021.04.022 34015256 PMC8282725

[bib86] Shime, H., M. Odanaka, M. Tsuiji, T. Matoba, M. Imai, Y. Yasumizu, R. Uraki, K. Minohara, M. Watanabe, A.J. Bonito, . 2020. Proenkephalin^+^ regulatory T cells expanded by ultraviolet B exposure maintain skin homeostasis with a healing function. Proc. Natl. Acad. Sci. USA. 117:20696–20705. 10.1073/pnas.2000372117 32769209 PMC7456133

[bib87] Singer, B.D., J.R. Mock, N.R. Aggarwal, B.T. Garibaldi, V.K. Sidhaye, M.A. Florez, E. Chau, K.W. Gibbs, P. Mandke, A. Tripathi, . 2015. Regulatory T cell DNA methyltransferase inhibition accelerates resolution of lung inflammation. Am. J. Respir. Cell Mol. Biol. 52:641–652. 10.1165/rcmb.2014-0327OC 25295995 PMC4491142

[bib88] Song, X., D. Dai, X. He, S. Zhu, Y. Yao, H. Gao, J. Wang, F. Qu, J. Qiu, H. Wang, . 2015. Growth factor FGF2 cooperates with interleukin-17 to repair intestinal epithelial damage. Immunity. 43:488–501. 10.1016/j.immuni.2015.06.024 26320657

[bib89] Spallanzani, R.G., D. Zemmour, T. Xiao, T. Jayewickreme, C. Li, P.J. Bryce, C. Benoist, and D. Mathis. 2019. Distinct immunocyte-promoting and adipocyte-generating stromal components coordinate adipose tissue immune and metabolic tenors. Sci. Immunol. 4:eaaw3658. 10.1126/sciimmunol.aaw3658 31053654 PMC6648660

[bib90] Tang, T.T., J. Yuan, Z.F. Zhu, W.C. Zhang, H. Xiao, N. Xia, X.X. Yan, S.F. Nie, J. Liu, S.F. Zhou, . 2012. Regulatory T cells ameliorate cardiac remodeling after myocardial infarction. Basic Res. Cardiol. 107:232. 10.1007/s00395-011-0232-6 22189560

[bib91] Togashi, Y., K. Shitara, and H. Nishikawa. 2019. Regulatory T cells in cancer immunosuppression - implications for anticancer therapy. Nat. Rev. Clin. Oncol. 16:356–371. 10.1038/s41571-019-0175-7 30705439

[bib92] Villalta, S.A., W. Rosenthal, L. Martinez, A. Kaur, T. Sparwasser, J.G. Tidball, M. Margeta, M.J. Spencer, and J.A. Bluestone. 2014. Regulatory T cells suppress muscle inflammation and injury in muscular dystrophy. Sci. Transl. Med. 6:258ra142. 10.1126/scitranslmed.3009925 PMC4889432 25320234

[bib93] Wang, J., L. Xie, C. Yang, C. Ren, K. Zhou, B. Wang, Z. Zhang, Y. Wang, K. Jin, and G.Y. Yang. 2015. Activated regulatory T cell regulates neural stem cell proliferation in the subventricular zone of normal and ischemic mouse brain through interleukin 10. Front. Cell. Neurosci. 9:361. 10.3389/fncel.2015.00361 26441532 PMC4568339

[bib94] Weirather, J., U.D. Hofmann, N. Beyersdorf, G.C. Ramos, B. Vogel, A. Frey, G. Ertl, T. Kerkau, and S. Frantz. 2014. Foxp3+ CD4+ T cells improve healing after myocardial infarction by modulating monocyte/macrophage differentiation. Circ. Res. 115:55–67. 10.1161/CIRCRESAHA.115.303895 24786398

[bib95] Westendorf, A.M., D. Fleissner, L. Groebe, S. Jung, A.D. Gruber, W. Hansen, and J. Buer. 2009. CD4+Foxp3+ regulatory T cell expansion induced by antigen-driven interaction with intestinal epithelial cells independent of local dendritic cells. Gut. 58:211–219. 10.1136/gut.2008.151720 18832523

[bib96] Winer, S., Y. Chan, G. Paltser, D. Truong, H. Tsui, J. Bahrami, R. Dorfman, Y. Wang, J. Zielenski, F. Mastronardi, . 2009. Normalization of obesity-associated insulin resistance through immunotherapy. Nat. Med. 15:921–929. 10.1038/nm.2001 19633657 PMC3063199

[bib97] Xia, N., Y. Lu, M. Gu, N. Li, M. Liu, J. Jiao, Z. Zhu, J. Li, D. Li, T. Tang, . 2020. A unique population of regulatory T cells in heart potentiates cardiac protection from myocardial infarction. Circulation. 142:1956–1973. 10.1161/CIRCULATIONAHA.120.046789 32985264

[bib98] Xiong, S., R. Guo, Z. Yang, L. Xu, L. Du, R. Li, F. Xiao, Q. Wang, M. Zhu, and X. Pan. 2015. Treg depletion attenuates irradiation-induced pulmonary fibrosis by reducing fibrocyte accumulation, inducing Th17 response, and shifting IFN-γ, IL-12/IL-4, IL-5 balance. Immunobiology. 220:1284–1291. 10.1016/j.imbio.2015.07.001 26224246

[bib99] Zhang, C., L. Li, K. Feng, D. Fan, W. Xue, and J. Lu. 2017. “Repair” Treg cells in tissue injury. Cell. Physiol. Biochem. 43:2155–2169. 10.1159/000484295 29069643

[bib100] Zhang, Y., J. Lazarus, N.G. Steele, W. Yan, H.J. Lee, Z.C. Nwosu, C.J. Halbrook, R.E. Menjivar, S.B. Kemp, V.R. Sirihorachai, . 2020. Regulatory T-cell depletion alters the tumor microenvironment and accelerates pancreatic carcinogenesis. Cancer Discov. 10:422–439. 10.1158/2159-8290.CD-19-0958 31911451 PMC7224338

[bib101] Zouggari, Y., H. Ait-Oufella, L. Waeckel, J. Vilar, C. Loinard, C. Cochain, A. Récalde, M. Duriez, B.I. Levy, E. Lutgens, . 2009. Regulatory T cells modulate postischemic neovascularization. Circulation. 120:1415–1425. 10.1161/CIRCULATIONAHA.109.875583 19770391

