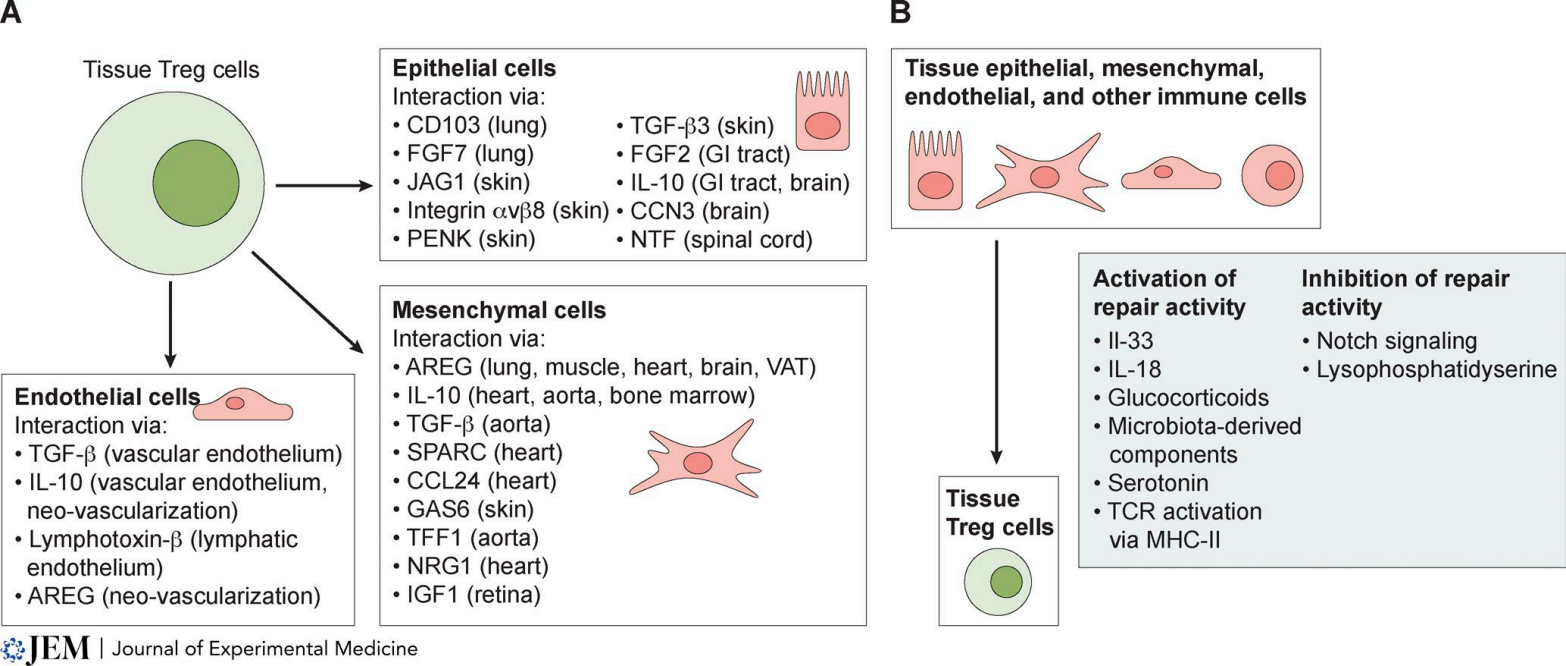# Correction: Treg–tissue cell interactions in repair and regeneration

**DOI:** 10.1084/jem.2023124405032024c

**Published:** 2024-05-13

**Authors:** Lucas F. Loffredo, Thomas M. Savage, Olivia R. Ringham, Nicholas Arpaia

Vol. 221, No. 6 | https://doi.org/10.1084/jem.20231244 | April 26, 2024

The authors regret that “TGF-β3” was accidentally written as “TGF-α3” in the “Epithelial cells” section of the originally published Fig. 2 A. The corrected figure is shown here. This correction does not change the original conclusions of the review, and the figure legend remains unchanged. The error appears in PDFs downloaded before May 3, 2024.

**Figure:**